# Oblique lumbar interbody fusion for adjacent segment disease after posterior lumbar fusion: a case-controlled study

**DOI:** 10.1186/s13018-019-1276-9

**Published:** 2019-07-16

**Authors:** Cong Jin, Minghua Xie, Lei He, Wenbin Xu, Weiqi Han, Wengqing Liang, Yu Qian

**Affiliations:** 1 Department of Orthopaedics, Shaoxing People’s Hospital (Shaoxing Hospital, Zhejiang University School of Medicine), Zhongxing North Road, Shaoxing, 312000 Zhejiang China; 2 Department of Orthopaedics, Sir Run Run Shaw Hospital, School of Medicine, Zhejiang University, Hangzhou, China

**Keywords:** Oblique, Lumbar interbody fusion, Adjacent segment disease

## Abstract

**Background:**

This study assessed clinical and radiographic outcomes of oblique lumbar interbody fusion (OLIF) in comparison with posterior reoperation for adjacent segment disease (ASD).

**Methods:**

A total of 26 patients with symptomatic ASD after lumbar fusion were included in this retrospective case-controlled study conducted from January 2013 to December 2018. Twelve patients underwent single-segment OLIF with or without posterior instrumentation (OLIF group), whereas 14 patients underwent posterior reoperation (posterior approach group). The clinical outcomes included operative time, blood loss, hospital stay, Visual Analogue Scale (VAS), Oswestry Disability Index (ODI), and complications. Preoperative and postoperative radiographic outcomes were compared.

**Results:**

The operative time (60.6 ± 16.1 min vs. 150.9 ± 28.5 min, respectively; *P* < 0.05) and the blood loss in the OLIF group 89.2 ± 49.0 ml vs. 340.7 ± 130.2 ml, respectively; *P* < 0.05) were significantly lower than those in the posterior group. The hospital stay was lower in the OLIF group than in the posterior approach group (6.6 ± 1.3 days vs. 9.5 ± 2.5 days, respectively; *P* < 0.05). In the posterior approach group, 6 of 14 patients (42.8%) had issue with dural tear, while none in the OLIF group had such issue (P < 0.05). The ODI score (13.2 ± 4.2 vs. 19.2 ± 7.2, respectively; *P* = 0.014) and the VAS back pain score were lower in the OLIF group postoperatively and at last follow-up. In the OLIF group, the radiographic outcomes were significantly improved postoperatively.

**Conclusions:**

Due to our results and early experiences, we proposed that OLIF was safe and effective for ASD. Compared with posterior reoperation, OLIF results in shorter operative time and hospital stay, lesser blood loss, and lower risk of dural injury.

## Background

Adjacent segment disease (ASD) is one of the most common complications of lumbar spine fusion [1]. Recently, spinal fusion has increasingly been used in the treatment of lumbar disorders with the development of surgical techniques and spinal instrumentation. In the USA, the annual incidence of spinal fusion has increased more than 600% during the last decade [2]. Spinal fusion techniques provide excellent clinical results for the treatment of various lumbar diseases; however, spinal fusion increases mechanical stress and segmental motion at adjacent segments, putting the patients at risk for developing ASD [3]. It has been reported that ASD was observed in 36–84% of patients at the 5-year follow-up after lumbar fusion on the basis of radiographic evidence [4, 5], and the incidence of symptomatic ASD requiring reoperation ranged from 5.2 to 18.5% [6].

Surgical intervention is considered when conservative therapies fail to relieve symptoms associated with ASD. There have been many surgical procedures described for the treatment of ASD, including the posterior approach with decompression and extended fusion [7], anterior lumbar interbody fusion (ALIF) [8], extreme lateral interbody fusion (XLIF) [9], and endoscopic surgery [10, 11]. Although most surgeons prefer the posterior approach for treating symptomatic ASD due to its effectiveness and familiarity, this approach is associated with a greater risk of dural tear and extensive surgical trauma to the paraspinal muscle [12, 13]. It has been reported that the incidence of dural tear during lumbar reoperation is estimated to be as high as 13.2–21.4%, whereas it was 7.6–10% during primary surgeries [13, 14]. Moreover, posterior reoperation may cause secondary damage to the paraspinal muscle, inducing postoperative back pain, muscle weakness, and functional disability at long-term follow-up [12]. Thus, anterior lumbar interbody fusion was recommended by some experts for the treatment of ASD with the advantages of less paraspinal muscle injury, low risk of operative dural tear, and less disturbance to nerve roots or cauda equina [15]; however, it also carries the risk of injury to the iliac vessels, ureter, and peritoneal content. To reduce the operative risks, a minimally invasive lateral approach such as XLIF was also applied to treat ASD [8]. However, XLIF involves blunt dissection of the psoas major muscle, which possibly leads to lumbar plexus injury. It has been reported that 30% of patients experience paresthesias in the leg, while 27% of patients experience thigh pain after XLIF [16]. More recently, endoscopic surgeries, including percutaneous endoscopic lumbar discectomy (PELD) and transforaminal endoscopic surgery, for ASD have been reported. For instance, Sun et al. reported that PELD resulted in shorter operation time, lesser blood loss, and faster recovery than posterior reoperation did [11]. However, some authors take issue with endoscopic surgeries for ASD. For instance, Telfeian reported that the 2-year failure rate is 33%, indicating the benefit of endoscopic surgery in patients with ASD may ultimately be temporary [10]. Therefore, the surgical treatment of ASD is a topic of continuing research.

Oblique lumbar interbody fusion (OLIF) was first described by Michael Mayer in 1977 [17], which involves accessing the disc space via an anterior approach between the aorta and psoas muscle. Lesser nerve injury has been reported for OLIF than for XLIF as the psoas muscle is not dissected or traversed in OLIF [18]. Moreover, Silvestre et al. reported good results with minimal blood loss, short operation time, and excellent functional rehabilitation achieved in 179 patients after OLIF [19]. However, to the best of our knowledge, clinical outcomes of OLIF for the treatment of ASD have not been published. Thus, we conducted a retrospective case-controlled study with a series of patients with symptomatic ASD who underwent OLIF or posterior reoperation. In this study, we aimed to assess the clinical and radiographic outcomes of OLIF in comparison with posterior reoperation.

## Materials and methods

### Study design

This study was a retrospective case-controlled study conducted at Shaoxing People’s Hospital from January 2013 to December 2018. Data were analyzed after obtaining approval from the Medical Ethics Committee of Shaoxing People’s Hospital (NO2019012) on January 15, 2019. Informed consent was obtained from all individual participants included in this study. The surgeries were performed by two senior surgeons. The radiographic evaluations were performed by two independent senior radiologists, and the final result was the average of the two testing values.

### Patients

The inclusion criteria for this study were single-level symptomatic ASD with failed conservative treatment for more than 3 months, age 18–65 years, MRI images showing inclusive disc herniation or mild degenerative spondylolisthesis (I or II) [20] at the adjacent segment, primary lumbar fusion treatment for degenerative diseases, and follow-up of more than 12 months after reoperation. Symptomatic ASD refers to the presence of clinical symptoms and signs occurring at the adjacent segment. The exclusion criteria included patients with images showing lumbar disc protrusion, severe degenerative spondylolisthesis (III or IV) [20], severe osteoporosis, and primary surgery for nondegenerative diseases including trauma, tumor, infection, or inflammation.

There were 26 patients with symptomatic ASD after posterior lumbar interbody fusion (PLIF) or transforaminal lumbar interbody fusion (TLIF) included in this study, of which 12 patients underwent single-segment OLIF with or without posterior internal fixation (OLIF group) and 14 patients underwent posterior approach with extended fusion and decompression (posterior approach group). Demographic data including age, sex, body mass index (BMI), bone mineral density (BMD), number of initial fusion segments, level of ASD, time to reoperation, and follow-up time were recorded.

### Procedure

In the OLIF group, patients were placed in the lateral decubitus position on their right side after the induction of general anesthesia. Under fluoroscopy, the center of the targeted disc was marked on the skin. A 4-cm skin incision was made 4–6 cm anterior to the center of the marked disc. External oblique, internal oblique, and transverse abdominal muscles were then dissected along the direction of their fibers in this muscle-splitting approach. Then, the retroperitoneal space was accessed by blunt dissection, and the peritoneal content was mobilized anteriorly. The psoas muscle was identified and retracted posteriorly. After the targeted disc was exposed and fluoroscopically verified, subtotal discectomy was performed and vertebral endplates were prepared. Afterwards, a cage (Clydesdale Spinal System, Medtronic, Inc., Minneapolis, MN, USA) filled with bone allograft was inserted, and its position was fluoroscopically confirmed. Rubber drains were placed, and the incision was closed in a layered fashion. For patients with preoperative BMD *<* − 1.0, additional posterior internal fixations were performed.

In the posterior approach group, a posterior midline incision was made. Partial laminectomy and partial facetectomy were performed for decompression. Then, discectomy and endplate preparation were performed. An interbody fusion cage (Medtronic, Inc., Memphis, TN, USA) filled with autograft bone was inserted into the intervertebral space. Initial rods were removed, and extended internal fixation with pedicle screw system (Medtronic, Inc., Memphis, TN, USA) was applied.

All patients were allowed weight-bearing for 3 days after surgery, and they wore a lumbar brace for 3 months postoperatively.

### Clinical evaluation

The clinical data, including operative time, blood loss, and length of hospital stay, were obtained. The perioperative complications, including incision infection, dural tear, nerve injury, ureteral injury, peritoneal content injury, cage displacement, and reoperation, were also recorded. The Oswestry Disability Index (ODI) [21] for low back pain was recorded preoperatively, 3 months postoperatively, and at the last follow-up. Leg and back pain evaluation using the Visual Analogue Scale (VAS) [22] was performed preoperatively, postoperatively, 3 months postoperatively, and at the final follow-up.

### Radiographic evaluation

**Fig. 1:**
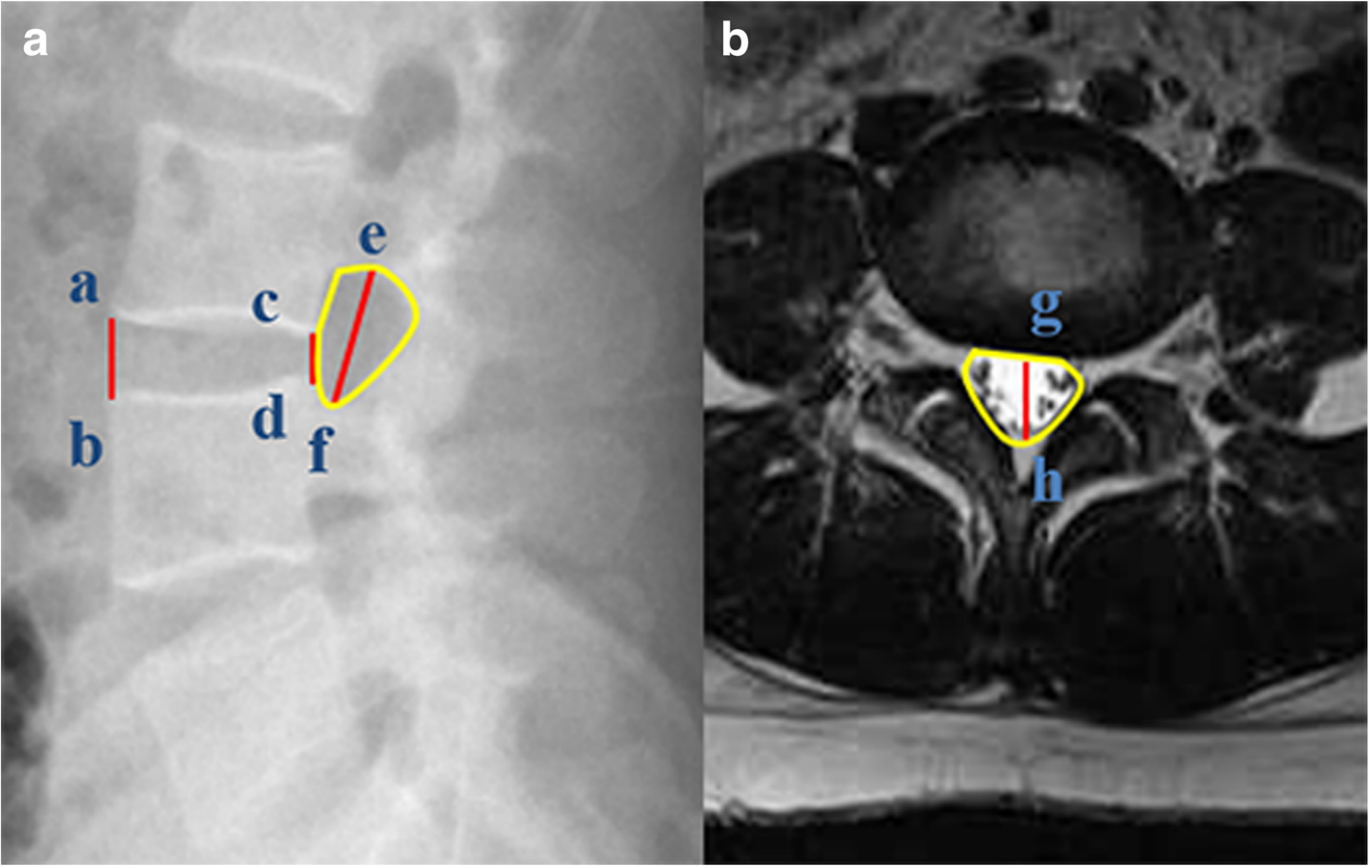
**a** The distance between point a and point b is the anterior height of the intervertebral space (*D*_1_). The distance between point c and point d is the posterior height of the intervertebral space (*D*_2_). IVH = (*D*_1_ + *D*_2_)/2. Point e is the peak of the inferior margin of the superior vertebral pedicle, and point f is the lowest point of the inferior margin of the superior vertebral pedicle. The distance between point e and point f is termed as the IFH. The area inside the region bounded by the yellow line is the IFA. **b** Point g is the midpoint of the anterior margin of the thecal sac. Point h is the midpoint of the posterior margin of the thecal sac. The distance between point g and point h is termed as the APD. The area inside the region bounded by the yellow line is the CSA

Lateral radiography of the lumbar spine was performed preoperatively and postoperatively to measure the IVH, intervertebral foraminal height (IFH), and intervertebral foraminal area (IFA) at the targeted segment (Fig. 1a). The IVH is defined as the average of the anterior and posterior heights of the intervertebral space. The IFH is defined as the maximum distance between the inferior margin of the superior vertebral pedicle and the superior margin of the inferior vertebral pedicle. The IFA is the area bounded by the vertebral posterior margin, inferior margin of the superior vertebral pedicle, and superior margin of the inferior vertebral pedicle. The anteroposterior diameter (APD) and cross-sectional area (CSA) of the thecal sac at the midline of the targeted intervertebral space were evaluated by T2-weighted MRI (Fig. 1b) preoperatively and postoperatively. All the radiographic measurements were evaluated using a digital measuring tool of the radiographic imaging system.

### Statistical analysis

Continuous variables were measured as mean ± standard deviation, and categorical variables were expressed as frequency or percentages. Independent *t* tests were used to compare demographic data including age, BMI, BMD, time to reoperation, and follow-up time between the two groups. Fisher’s exact test and *χ*^2^ test were used to examine the differences in sex, number of fusion segments, level of ASD, and complications between the two groups. Paired *t* tests were used to compare the ODI score, VAS score, and radiographic parameters preoperatively and postoperatively. Independent *t* tests were used to compare the ODI score, VAS score, and radiographic parameters between the two groups. Shapiro-Wilk test was used to ensure the normal distribution before *t* test conducted. SPSS software (version 19.0, IBM Corp., Armonk, NY, USA) was used for statistical analysis. The level of significance was set at *P* < 0.05.

## Results

### Demographic data

From January 2013 to December 2018, a total of 26 patients were enrolled in this retrospective study. There were 12 patients in the OLIF group and 14 patients in the posterior approach group. In the OLIF group, 9 patients underwent OLIF without posterior internal fixation, while 3 patients underwent OLIF with posterior instrumentation. There were no statistically significant differences between the OLIF and posterior approach groups in terms of age, sex, BMI, BMD, number of initial fusion segments, level of ASD, time to reoperation, and follow-up time (Tables 1 and 2).

**Table 1:** Demographic information

	OLIF group	Posterior approach group	*P*
Number	12	14	
Age (years)	53.4 ± 7.2	53.9 ± 7.4	0.879
Sex (M/F)	3/9	8/6	0.098
BMI (kg/m^2^)	23.9 ± 2.1	24.8 ± 1.7	0.223
BMD	0.3 ± 1.5	1.0 ± 1.6	0.208
Time to reoperation (years)	6.8 ± 3.4	8.4 ± 3.7	0.261
Number of initial fusion segments			
One	5	2	0.289
Two	6	10	
Three	1	2	
Level of ASD			
L1/2	0	1	0.589
L2/3	4	2	
L3/4	6	8	
L4/5	2	2	
L5/S1	0	1	
Follow-up time (months)	15.5 ± 3.4	16.4 ± 4.2	0.541

**Table 2:** Patient list

Patient no.	Age	Sex	Initial fusion segment	Time to reoperation (years)	Level of ASD	Reoperation
1	51	Male	L4–S1	5	L3/4	TLIF
2	53	Male	L2–L5	7	L1/2	PLIF
3	55	Female	L4–S1	10	L3/4	PLIF
4	61	Female	L3–S1	14	L2/3	TLIF
5	42	Female	L5–S1	5	L4/5	TLIF
6	45	Male	L4–L5	5	L3/4	OLIF
7	51	Male	L4–S1	8	L3/4	TLIF
8	48	Female	L3–L5	4	L2/3	OLIF
9	45	Male	L3–L5	4	L5/S1	PLIF
10	51	Female	L4–S1	7	L3/4	OLIF
11	58	Male	L4–S1	11	L3/4	PLIF
12	41	Female	L5–S1	5	L4/5	OLIF
13	64	Female	L3–L5	13	L2/3	OLIF + posterior instrumentation
14	51	Female	L4–S1	6	L3/4	PLIF
15	63	Male	L3–L5	11	L2/3	PLIF
16	64	Female	L4–S1	12	L3/4	PLIF
17	52	Female	L4–L5	2	L3/4	OLIF
18	65	Male	L4–S1	15	L3/4	TLIF
19	45	Male	L5–S1	4	L4/5	TLIF
20	62	Male	L4–S1	12	L3/4	OLIF + posterior instrumentation
21	50	Female	L4–S1	6	L3/4	TLIF
22	56	Female	L3–S1	6	L2/3	OLIF + posterior instrumentation
23	55	Male	L4-L5	4	L3/4	OLIF
24	61	Female	L3-L5	6	L2/3	OLIF
25	58	Female	L4-S1	10	L3/4	OLIF
26	48	Female	L5-S1	8	L4/5	OLIF

### Clinical outcomes

The OLIF group was superior to the posterior approach group in terms of the operative time, blood loss, and length of hospital stay. The operative time of the OLIF group was significantly shorter than that of the posterior approach group (60.6 ± 16.1 min vs. 150.9 ± 28.5 min, respectively; *P* = 0.000). The blood loss in the OLIF group (89.2 ± 49.0 ml) was significantly lower than that in the posterior approach group (340.7 ± 130.2 ml, *P* = 0.000). The hospital stay was 6.6 ± 1.3 days in the OLIF group and 9.5 ± 2.5 days in the posterior approach group (*P* = 0.001) (Table 3).

**Table 3:** Clinical data

	OLIF group	Posterior approach group	*P*
Operative time (min)	60.6 ± 16.1	150.9 ± 28.5	0.000
Blood loss (ml)	89.2 ± 49.0	340.7 ± 130.2	0.000
Hospital stay (days)	6.6 ± 1.3	9.5 ± 2.5	0.001
Perioperative complications	2 (16.7%)	7 (50.0%)	0.075
Incision infection	0	1 (7.1%)	0.345
Dural tear	0	6 (42.8%)	0.010
Nerve injury	1 (8.3%)	0	0.271
Ureteral injury	0	0	
Vascular injury	0	0	
Peritoneal content injury	0	0	
Cage subsidence	1 (8.3%)	0	0.271
Reoperation	1 (8.3%)	0	0.271

Overall, the incidence of perioperative complications in the OLIF group was comparable to that of the posterior approach group (*P* = 0.075). In the posterior approach group, 6 out of 14 patients (42.8%) had issues with the complication of dural tear, whereas none in the OLIF group had issues; this difference was statistically significant (*P* = 0.010). Incision infection occurred in 1 out of 14 (7.1%) patients in the posterior approach group, which was successfully treated with dressings and oral antibiotics. In the OLIF group, lumbar plexus injury occurred in one patient with a clinical presentation of transient numbness and left leg pain; fortunately, the patient recovered spontaneously 3 months postoperatively. Furthermore, 1 out of 12 patients (8.3%) in the OLIF group required posterior instrumentation as a result of cage subsidence (Table 3).

**Fig. 2:**
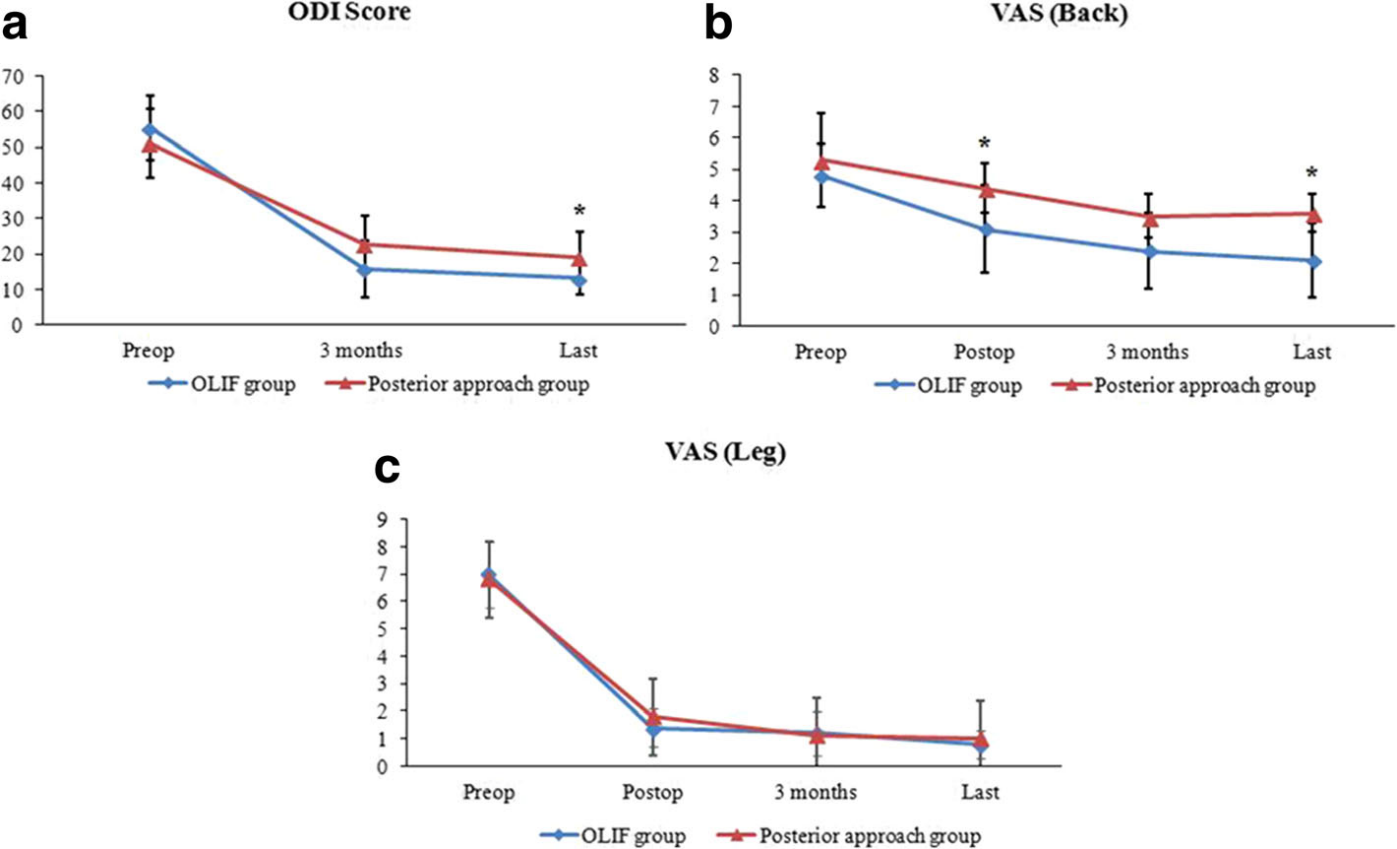
**a** ODI scores of the two groups preoperatively, 3 months postoperatively, and at the last follow-up. **b** VAS back pain scores of the two groups preoperatively, postoperatively, 3 months postoperatively, and at the last follow-up. **c** VAS leg pain scores of the two groups preoperatively, postoperatively, 3 months postoperatively, and at the last follow-up. *P < 0.05 when compared with the OLIF group

The OLIF group was superior to the posterior approach group in terms of ODI score and VAS back pain score. The ODI score in the OLIF group improved from 55.1 ± 8.1 preoperatively to 13.2 ± 4.2 at the last follow-up with a significant difference (*P* = 0.000). ODI scores were lower in the OLIF group than in the posterior approach group at the last follow-up (13.2 ± 4.2 vs. 19.2 ± 7.2; *P* = 0.014). The VAS back pain score was significantly lower in the OLIF group than in the posterior approach group postoperatively (3.2 ± 0.8 vs. 5.3 ± 1.0; *P* = 0.040). In addition, the VAS back pain score in the OLIF group was 2.1 ± 0.7, which was also significantly lower than 3.6 ± 1.2 in the posterior approach group at the last follow-up (*P* = 0.015). The VAS leg pain score in the OLIF group improved from 7.0 ± 1.1 preoperatively to 1.3 ± 0.9 postoperatively with a significant difference (*P* = 0.000). There were no significant differences in the VAS leg pain score between the two groups postoperatively, 3 months postoperatively, and at the last follow-up (Fig. 2).

**Fig. 3:**
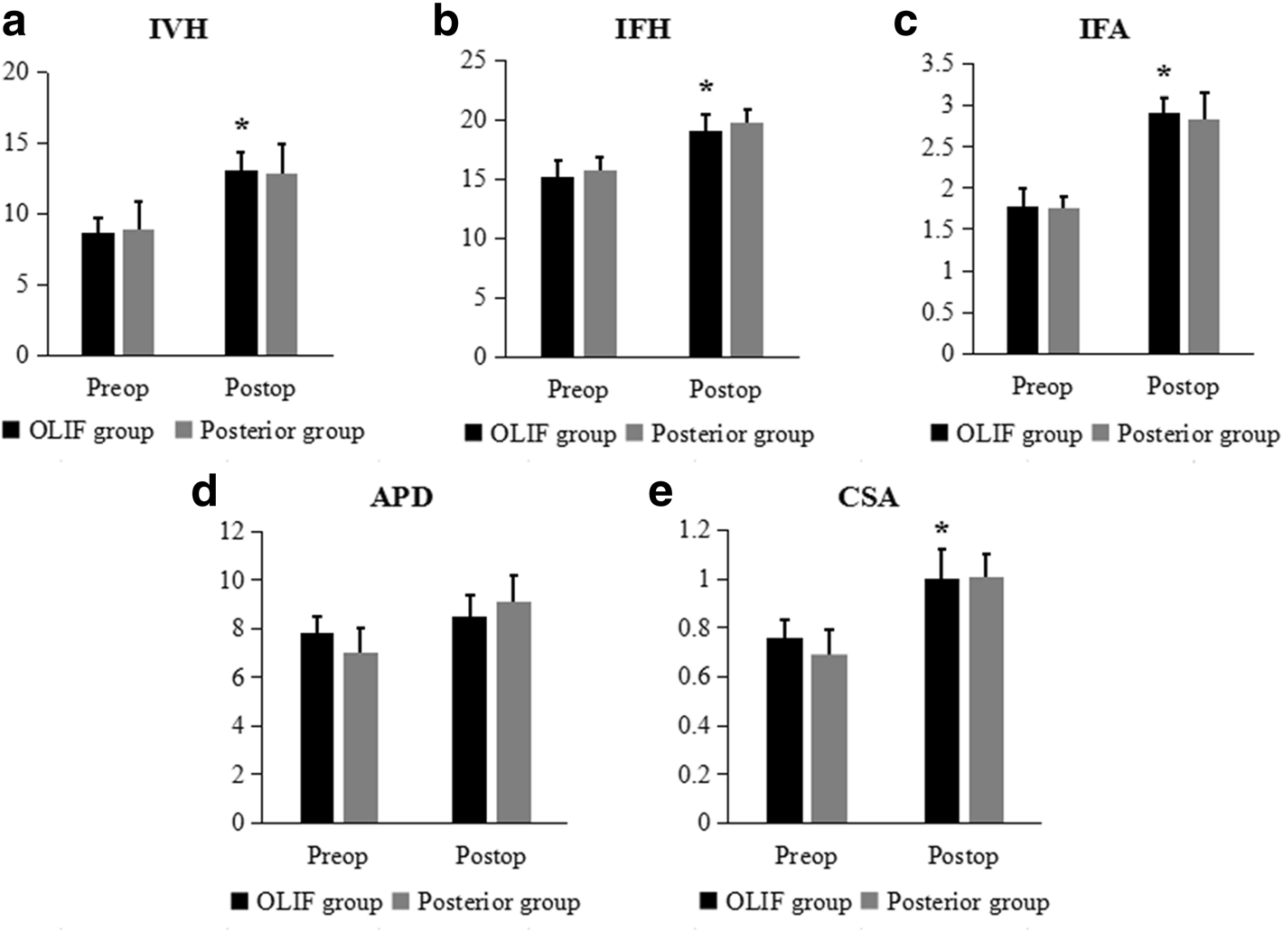
**a** IVH, **b** IFH, **c** IFA, **d** APD, and **e** CSA of the two groups preoperatively and postoperatively. *P < 0.05 when compared with the OLIF group preoperatively

**Fig. 4:**
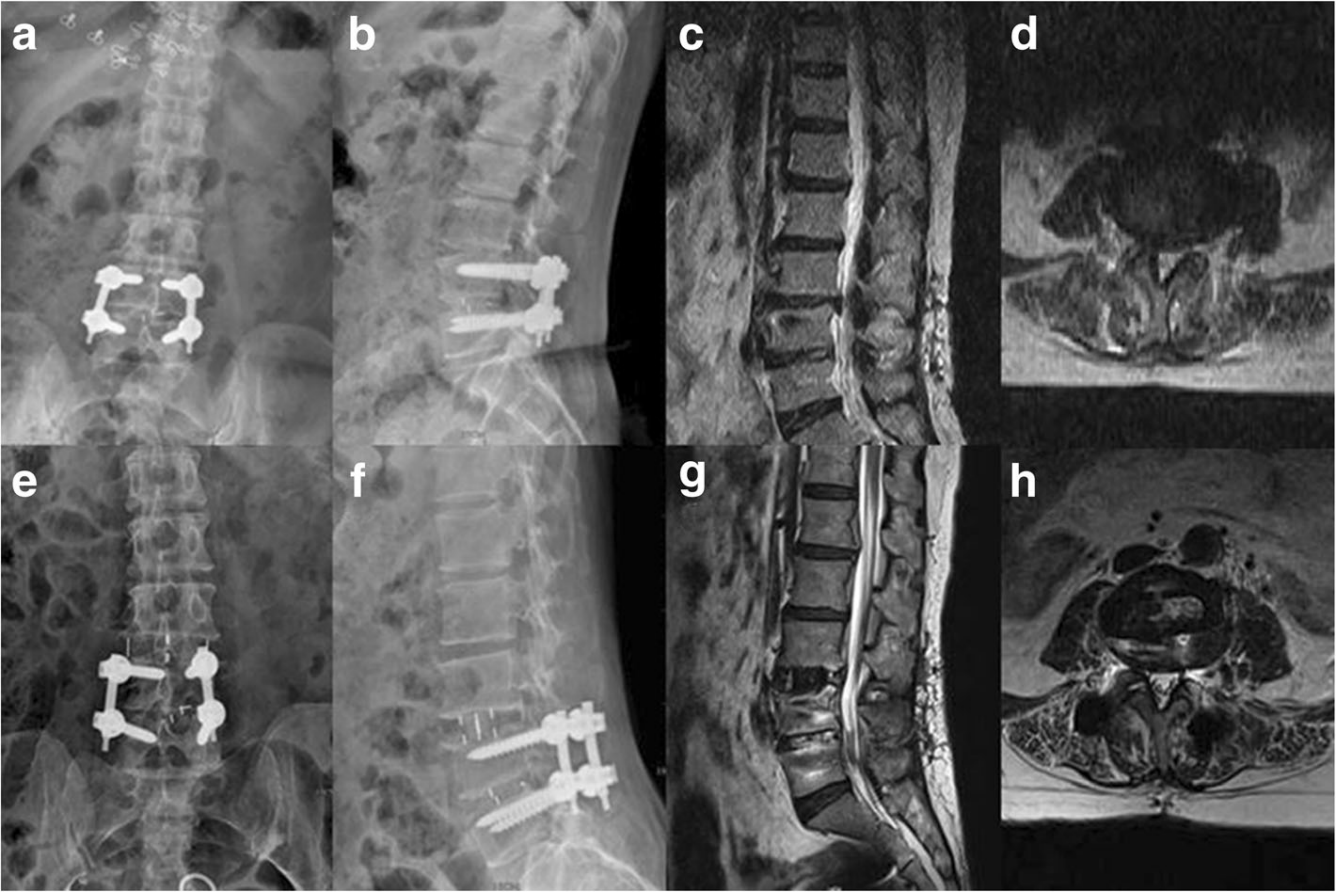
A 52-year-old female with a symptomatic ASD at the L3/4 level. Primary transforaminal lumbar interbody fusion was performed to treat the disc herniation at L4/5 2 years ago. OLIF without posterior instrumentation was employed to treat ASD at L3/4. **a**, **b** Preoperative X-ray shows loss of intervertebral disc height at L3/4, and posterior instrumentation and an intervertebral cage were placed appropriately at L4/5. Preoperatively, the IVH was 8.9 mm, the IFH was 15.4 mm, and the IFA was 1.76 cm^2^. **c**, **d** Preoperative MRI shows inclusive disc herniation at L3/4 and compression of right L4 nerve root. No obvious proliferation or calcification of the ligamentum flavum or zygopophysis was noted. Preoperatively, the APD was 6.6 mm and the CSA was 0.70 cm^2^. **e**, **f** Postoperative X-ray shows the intervertebral cage at L3/4 was well in place, and the IVH, IFH, and IFA were significantly increased. Postoperatively, the IVH was 14.8 mm, the IFH was 18.8 mm, and the IFA was 2.85 cm^2^. **g**, **h** Postoperative MRI shows the spinal canal was apparently enlarged and effective indirect decompression was achieved. The APD was improved from 6.6 mm preoperatively to 8.6 mm postoperatively, and the CSA postoperatively was 0.92 cm^2^, which was apparently larger than 0.70 cm^2^ preoperatively

The radiographic outcomes were significantly improved postoperatively in the OLIF group, which were comparable to those of the posterior approach group. In the OLIF group, the IVH was 8.7 ± 1.0 mm preoperative and 13.1 ± 1.0 mm postoperative (*P* = 0.000). The IFH was significantly higher postoperatively than preoperatively in the OLIF group (19.1 ± 1.4 mm vs. 15.2 ± 1.4 mm; *P* = 0.000). Furthermore, the IFA was 2.91 ± 0.19 cm^2^ postoperatively, which was also significantly larger than 1.77 ± 0.23 cm^2^ preoperatively (*P* = 0.000). The postoperative APD was 8.5 ± 0.9 mm, which was higher than 7.8 ± 0.7 mm preoperatively; however, the difference was not statistically significant (*P* = 0.053). Moreover, the CSA was significantly larger postoperatively than preoperatively (1.00 ± 0.12 cm^2^ vs. 0.75 ± 0.07 cm^2^, respectively; P = 0.000). All the radiographic outcomes, including the IVH, IFH, IFA, APD, and CSA, were comparable between the two groups postoperatively (*P* > 0.05) (Figs. 3 and 4).

## Discussion

Although ASD is a common complication of spinal fusion, only few studies have investigated the clinical outcomes of surgical treatment for ASD. OLIF is one type of lateral lumbar interbody fusion (LLIF), and access the disc space via an anterolateral approach between the aorta and psoas muscle. LLIF was recommended by some experts for the treatment of ASD with less postoperative pain and favorable radiographic outcomes [23]. Moreover, Aichmair et al. reported LLIF was an effective surgical treatment option for ASD with satisfactory clinical results achieved in 53 patients [9]. In this study, we reported that the OLIF technique was used effectively and safely for the treatment of ASD, and compared with the conventional posterior reoperation for ASD, OLIF resulted in shorter operative time and hospital stay, lesser blood loss, lower risk of dural injury, and lower incidence of postoperative back pain.

The results showed that during OLIF, blood loss was apparently lesser and the operative time shorter compared with those during posterior approaches. In this study, the blood loss and operative time were 89.2 ± 49.0 ml and 60.6 ± 16.1 min, respectively, in the OLIF group; this was consistent with the findings of previous studies. For instance, Silvestre et al. reported that the OLIF procedure was associated with minimal blood loss (57 ± 131 ml) and a short operation time (32.5 ± 13.2 min) in 179 patients [19]. This may be due to the fact that a smaller surgical incision made by the muscle-splitting approach in the OLIF technique reduced the risk for injury in the surrounding tissues, resulting in less bleeding. Furthermore, compared with the posterior approach, OLIF causes fewer disturbances to the spinal canal and helps avoid posterior scar tissues, leading to lesser blood loss and shorter operative time. In OLIF, interbody fusion was performed using a bone substitute without harvesting an autogenous bone graft, which can potentially decrease surgical time. In the present study, the average length of hospital stays was only 6.6 ± 1.3 days in OLIF, which was significantly shorter than that of the posterior approach. This indicates that patients who underwent OLIF had a better rehabilitation, and OLIF could potentially reduce the consumption of clinical resources and thus decrease the cost of disease treatment.

Another finding of this study was that the complication of dural tear in OLIF was apparently lower than that in the posterior approach, which is an important advantage of OLIF. In the current study, we approached the disc between the aorta and the psoas muscle in the OLIF group, and consequently, the occurrence of lumbar plexus injury decreased compared with XLIF. Nevertheless, one patient presented with transient numbness and pain on the left leg that resolved spontaneously within 3 months after surgery. In this study, 1 out of 12 patients (8.3%) required posterior reoperation as a result of cage subsidence; this was similar with the findings of previous studies. For example, Ohtori et al. reported that the reoperation rate of stand-alone OLIF was 9.5% [24]. Some reports also showed that cage subsidence or endplate injury was the leading complication of stand-alone OLIF, ranging from 13.5 to 18.7% [18]. Thus, initial posterior fixation was required to avoid revision surgery or cage subsidence if endplate injury was suspected intraoperatively.

The findings showed that the OLIF group demonstrated better clinical improvement in terms of ODI and VAS back pain scores in the early postoperative period compared with the posterior approach group. In previous studies, clinical improvement in leg pain following OLIF was observed with a 3.2–7.2 score reduction in the VAS score, while an improvement in lower back pain was quantified by a 34–50 point reduction in the ODI score [24]. This may be attributed to the minimally invasive nature of OLIF. The OLIF technique preserves posterior anatomical structures including the facet, lamina, paraspinal muscles, and ligamentous structures. Secondary injury to posterior structures and extensive stripping of the paraspinal muscles after posterior reoperation surgery for ASD can lead to additional problems, such as back stiffness, chronic back pain, and slow postoperative recovery [7]. The preservation of posterior anatomical structures was also an important advantage of the OLIF technique over the posterior approach.

In this study, effective decompression was achieved by OLIF for the treatment of ASD. In OLIF, an intervertebral cage with a larger area and volume can be implanted, which can effectively restore the disc space and intervertebral foramen height, thus allowing indirect spinal canal decompression. It has been reported that the average CSA of the thecal sac increased from 99.6 mm^2^ preoperatively to 134.3 mm^2^ postoperatively in OLIF [25]. Moreover, Fujibayashi et al. also reported that OLIF was associated with a 19.0–33.1% increase in cross-sectional thecal sac area and 24.7% increase in the IFA on MRI [26]. Similar to previous research, in this study, the IFA and cross-sectional thecal sac area were significantly increased postoperatively in the OLIF group. However, if patients have severe spinal central stenosis caused by facet hypertrophy, thickening of the ligamentum flavum, or calcification of disc herniation, a posterior reoperation including PLIF or TLIF should be considered.

There are several limitations to the current study. First, the study was conducted retrospectively by case selection and was not randomized and controlled. Second, the duration of follow-up was short and the sample size was relatively small. Nevertheless, future prospective randomized studies involving a long-term follow-up with a larger number of patients are needed to elucidate the advantages of OLIF over posterior reoperation for ASD treatment.

## Conclusions

Due to our results and early experiences, we proposed that OLIF was safe and effective for ASD. Furthermore, compared to posterior reoperation, OLIF results in shorter operative time and hospital stay, lesser blood loss and lower risk of dural injury.

## Data Availability

The authors declare that all data supporting the findings of this study are available within the article.

## Abbreviations

ALIF: Anterior lumbar interbody fusion
APD: Anteroposterior diameter
ASD: Adjacent segment disease
BMD: Bone mineral density
BMI: Body mass index
CSA: Cross-sectional area
IFA: Intervertebral foraminal area
IFH: Intervertebral foraminal height
IVH: Intervertebral height
LLIF: Lateral lumbar interbody fusion
ODI: Oswestry Disability Index
OLIF: Oblique lumbar interbody fusion
PELD: Percutaneous endoscopic lumbar discectomy
PLIF: Posterior lumbar interbody fusion
TLIF: Transforaminal lumbar interbody fusion
VAS: Visual Analogue Scale
XLIF: Extreme lateral interbody fusion
